# Intravenous methylprednisolone pulse therapy and the risk of in-hospital mortality among acute COVID-19 patients: Nationwide clinical cohort study

**DOI:** 10.1186/s13054-023-04337-5

**Published:** 2023-02-08

**Authors:** Takuhiro Moromizato, Ryoto Sakaniwa, Yasuharu Tokuda, Kiyosu Taniguchi, Kenji Shibuya

**Affiliations:** 1 Okinawa Nanbu Prefectural Medical Center and Children’s Medical Center, Haebaru, Okinawa Japan; 2 Department of Social Medicine, Osaka University Graduate School of Medicine, Suita, Osaka Japan; 3 Faculty of Medicine, University of Tsukuba, Tsukuba, Ibaraki Japan; 4 Tokyo Foundation for Policy Research, Minato-ku, Tokyo Japan; 5 Muribushi Okinawa Center for Teaching Hospitals, Urasoe, Okinawa Japan; 6 National Hospital Organization Mie National Hospital, Tsu-shi, Mie Japan

**Keywords:** COVID-19, Steroid pulse therapy, Critical care, Mechanical ventilation, In-hospital mortality

## Abstract

**Background:**

Steroids are widely used to modulate the inflammatory reactions associated with coronavirus disease 2019 (COVID-19); however, the optimal upper limit dose of steroid use for acute COVID-19 care remains unclear and currently available data may suffer from a time-dependent bias of no effectiveness or reversed causation given the desperate situation of treatment during this pandemic. Accordingly, the aim of this study was to elucidate the impact of intravenous pulse therapy with methylprednisolone (500 mg or greater per day) on the risk of in-hospital mortality among patients with COVID-19 by controlling for time-dependent bias.

**Methods:**

We performed a prospective cohort study with 67,348 hospitalised acute COVID-19 patients at 438 hospitals during 2020–2021 in Japan. The impact of intravenous methylprednisolone pulse therapy on the risk of in-hospital mortality was examined based on hazard ratios (HRs) and 95% confidence intervals (95% CIs), with stratification according to the status of invasive mechanical ventilation (iMV). Time-dependent bias was controlled for in a marginal structural model analysis, with reference to patients without methylprednisolone therapy.

**Results:**

During the study period, 2400 patients died. In-hospital mortality rates of iMV-free patients without or with methylprednisolone pulse therapy were 2.3% and 19.5%, and the corresponding values for iMV-receiving patients were 24.7% and 28.6%, respectively. The marginal structural model analysis showed that intravenous pulse therapy with methylprednisolone was associated with a lower risk of in-hospital mortality among patients receiving-iMV (HR 0.59; 95% CI 0.52–0.68). In contrast, pulse therapy with methylprednisolone increased the risk of in-hospital mortality among iMV-free patients (HR 3.38; 95% CI 3.02–3.79). The benefits of pulse therapy for iMV-receiving patients were greater than in those treated with intermediate/higher doses (40–250 mg intravenously) of methylprednisolone (HR 0.80; 95% CI 0.71–0.89).

**Conclusion:**

The results of our study suggest that intravenous methylprednisolone showed dose–response efficiencies, and pulse therapy may benefit critically ill patients with acute COVID-19, such as those requiring iMV.

**Supplementary Information:**

The online version contains supplementary material available at 10.1186/s13054-023-04337-5.

## Background

The coronavirus disease 2019 (COVID-19) pandemic has caused global devastation, causing several million deaths since 2019. The Randomised Evaluation of COVID-19 Therapy (RECOVERY) trial highlighted the effectiveness of dexamethasone (oral dose of 6 mg for up to 10 days) in reducing mortality in COVID-19 patients requiring oxygen [1]. Steroids are considered to modulate the inflammatory reactions associated with COVID-19. A higher dose and use of an intravenous route of administration, such as repeated-pulse therapy with intravenous methylprednisolone (500 mg or greater per day for three days), may be needed to suppress severe inflammation, including acute respiratory distress syndrome [2–4]. Notably, the upper limit of the steroid dose for hospitalised COVID-19 patients has not been sufficiently evaluated, and adverse impacts of high-dose steroids on mortality are not described in international guidelines [5]. Pulse dosing of steroids can potentially increase the risk of fatal complications such as aspergillosis/mucormycotic infections, and gastrointestinal, renal, hepatic, metabolic, or coagulation abnormalities [6, 7]. Indeed, previous meta-analyses and systematic reviews of observational studies have reported conflicting results regarding the outcomes of high doses of steroid therapy [5, 8, 9]. However, these results are likely affected by numerous confounders and biases. For example, some patients with desperate conditions may have received steroid pulse therapy despite being beyond the optimal timing of treatment, since the care teams or family members were looking at any option to save the patients, which would potentially lead to a per se “immortal time bias” or a time-dependent bias of no effectiveness or reversed causation [10]. Consequently, the optimal upper limit dose of steroid use for acute COVID-19 care remains unclear.

The marginal structural model, a recently developed statistical approach, allows for controlling the time-changing impacts of treatments and confounders on clinical outcomes; this form of modelling can address time-dependent bias, including the immortal time bias in observational studies [11, 12]. Therefore, we employed this statistical approach to evaluate the appropriateness of methylprednisolone pulse therapy and clarify its clinical implications for patients with critical conditions while reducing the immortal time bias. The data were obtained from a nationwide clinical cohort database of more than 60,000 acute COVID-19 patients in Japan, where steroid pulse therapy had been conventionally prescribed for acute inflammatory syndrome even before the first wave of the COVID-19 pandemic [13].

## Methods

### Study population

This study examined the diagnosis and procedure combination (DPC) database, a nationwide acute-care hospital administrative database in Japan. The DPC database was originally created by the Ministry of Health, Welfare, and Labour of Japan, and the data analysed in the current study were imported from the original national database compiled by medical data vision (MDV) Co., Tokyo, Japan [14]. Recent studies on COVID-19 outcomes have employed data from the DPC database provided by the MDV [15]. The age and sex distribution of patients registered in our DPC database are comparable to those of patients at nationwide healthcare institutes, which represent the national database and are officially published by the Japanese government [14]. Relatively high validity of primary diagnosis for DPC assessment has been reported previously, with 78.9% and 93.2% sensitivity and specificity, respectively [16].

The data analysed in the current study were obtained from 25% (438 of 1750) of all acute-care hospitals (DPC hospitals). From the database, we collected data for patients of all age ranges who were confirmed to have acute COVID-19 [International Classification of Diseases and Related Health Problems, 10th Revision (ICD-10) diagnosis code U071] with a positive reverse transcription-polymerase chain reaction test result. We included 67,348 patients who required admission due to acute COVID-19 between January 1, 2020, and November 30, 2021. The data from the first admission were used in the present study. The ethics committee of the Muribushi Okinawa Center for Teaching Hospitals approved the study protocol (No. 2021-9).

### Data collection

Individual patient data included information regarding demographic characteristics (age and sex), smoking history, body mass index, clinical status at admission (shock, coma, or cardiorespiratory failure), comorbidities, medications, treatment modalities, detailed timing of administered treatment modalities, and outcomes at discharge (in-hospital mortality). Data for comorbidities were collected using ICD-10 coding, based on a previous study that used MDV DPC data [17]. To deal with missing data for smoking history and body mass index, we applied multiple imputations with chained equations to include data from all available admitted patients [17]. Our data for treatment modalities included information regarding requirements for intensive care unit (ICU) admission, non-invasive positive pressure ventilation, invasive mechanical ventilation (iMV), oxygen therapy (oxygen provision by nasal cannula, mask, reservoir mask, or nasal high flow), and renal replacement therapy. For intravenous methylprednisolone therapy, a pulse dose was defined as a dose of 500 mg or greater of intravenous methylprednisolone prescribed per day. The intermediate or higher steroid dose was defined as a 40–250 mg dose of intravenous methylprednisolone prescribed per day. The impacts of 251–499 mg and 1–39 mg doses on mortality could not be investigated in the present study largely because the ampoule size of the methylprednisolone products is limited to the following four sizes in Japan: 40 mg, 125 mg, 500 mg, and 1000 mg (Additional file 1: Fig. S1).

### Statistical analyses

**Fig. 1:**
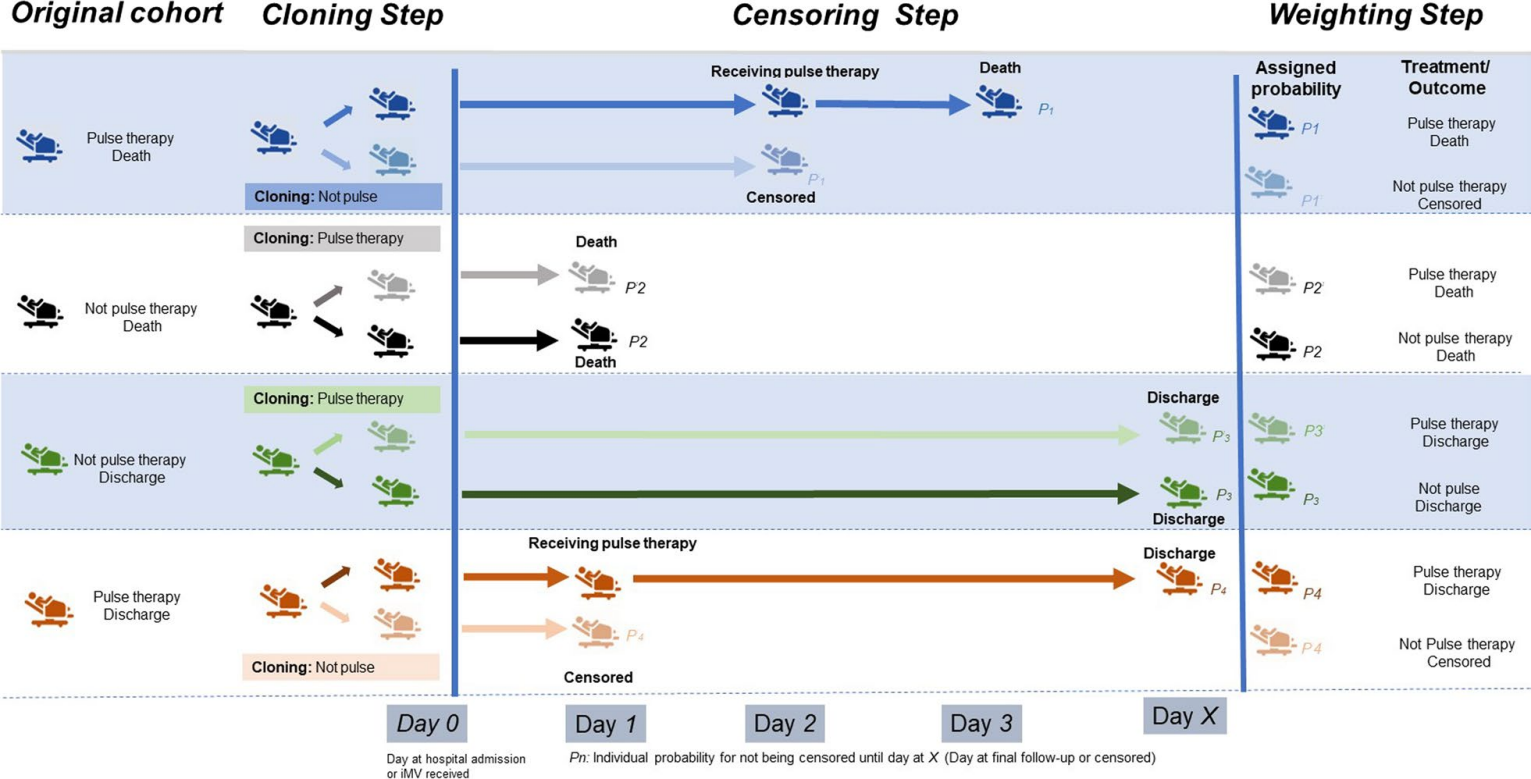
Summary of the cloning, censoring, and weighting method for analysis. In the cloning step, we created clones of each patient from the original cohort and assigned these clones to the opposite treatment (pulse therapy or no pulse therapy) from that of the original patient. In the censoring step, we censored follow-up according to discharge, death, or final follow-up, or when the original patients received pulse therapy (clone only). We also examined the individual probability for not being censored until the final follow-up (Day *X*). Such censoring is likely to be informative but could still lead to selection bias

**Fig. 2:**
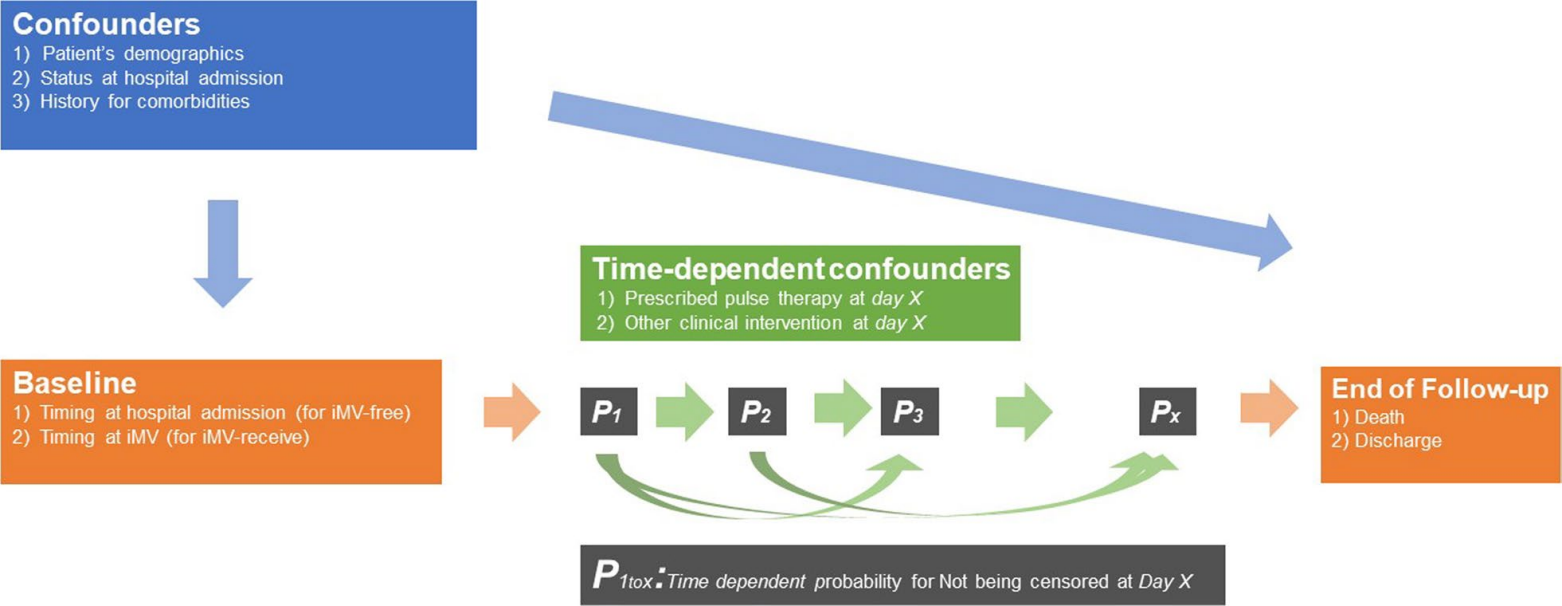
Causal graph of the marginal structural model analysis. The probability for not being censored at day *X* was estimated with baseline characteristics, status at hospital admission, history of comorbidities, and time before clinical intervention until day* X* – 1

All analyses were stratified according to iMV status: iMV-free or iMV-receiving. For the baseline characteristics, clinical interventions during admission, and the timing of interventions, continuous variables were described as mean and nominal variables as count and proportion. Cox proportional hazard models were used to examine the association between pulse therapy and the risk of in-hospital mortality. The models were constructed using age, sex, and history of comorbidities data. In multivariable adjustment, variables were selected based on the Bayesian information criterion score and its independence with similar variables. Specifically, variables that developed prior to the steroid therapy were introduced to the multivariate adjustment model. To eliminate immortal time bias and to control for time-dependent confounders such as time to other clinical interventions, we used cloning, censoring, and weighting to conduct a robust analysis based on the marginal structural model [12, 18–21]. The analysis methods are schematically described in Figs. 1 and 2, and more detailed information is described in the Supplementary Text (Additional file 2). Finally, we examined the hazard ratio (HR) and 95% confidence interval (CI) of in-hospital mortality for patients prescribed only intermediate/higher doses of steroids or those prescribed only methylprednisolone pulse therapy, including 66,418 of 67,348 patients, considering that 930 patients received both pulse therapy and intermediate or higher doses of methylprednisolone during the same admission period. In addition, we confirmed whether the gap between the initiation of iMV and the initiation of steroid pulse therapy was associated with the risk of in-hospital mortality in the restricted group. For sensitivity analyses, we also evaluated monthly variation in opportunities for methylprednisolone prescription and dose of the steroid. In addition, to further deal with the immortal time bias, we conducted time-dependent propensity score matching [22]. We also examined the influence of unmeasured confounders on the target association using the *E*-value test, which assesses the necessary impacts of unmeasured confounders to shift the level of target association to non-significant [23, 24]. All statistical analyses were performed using SAS 9.4 (SAS Inc. Carey, NC, USA). Statistical significance was set at *p* < 0.05.

## Results

### Patient characteristics

Among the 67,348 patients, 42.5% were female and the mean age (± standard deviation) was 53.7 (± 21.6) years. Intravenous pulse therapy with methylprednisolone was administered to 2244 (3.3%) patients, out of which 430 received iMV and 1814 did not receive iMV. A total of 2400 patients died during their hospital admissions. The in-hospital mortality rates of iMV-free patients without or with steroid pulse therapy were 2.3% and 19.5%, respectively, while the corresponding values for iMV-receiving patients were 24.7% and 28.6%, respectively. The median (interquartile range) first-day methylprednisolone doses were 1000 (500–1000) mg per day for pulse treatment and 80 (40–250) mg per day for intermediate/higher dose therapy. During the study period, a calendar effect was not confirmed regarding the opportunities for methylprednisolone prescription and their doses (Additional file 1: Table S1).

The differences in baseline characteristics (demographics, status on admission, and comorbidities) of hospitalised acute COVID-19 patients at admission, which were stratified according to the use of iMV and steroid pulse therapy, are described in Table 1. A majority of patients were categorised as “iMV-free and not prescribed pulse therapy” (*n* = 63,149/67,348). These patients were younger, included a higher proportion of female patients, were healthier at admission, and had a lower prevalence of comorbidities. The major differences from the other three groups were a low prevalence of hospital transfer and hypertension.

**Table 1:** Baseline characteristics of COVID-19 patients

Variables	iMV-free patients		iMV-receiving patients	
Pulse methylprednisolone therapy (≥ 500 mg per day)^a^	Not prescribed	Prescribed	Not prescribed	Prescribed
*Demographics*				
Number at risk, n	63,149	1814	1955	430
Age, years	53.0	63.5	64.3	64.1
Male, %	56.5	70.2	75.1	71.9
Body mass index, kg/m^2^	24.0	25.5	26.2	26.8
Current or past smokers, %	41.1	48.8	55.7	49.1
*Status at hospital admissions*				
Coma, %	0.3	0.6	1.5	0.7
Shock, %	0.0	0.0	0.1	0.0
Cardio-respiratory failure, %	47.8	53.3	74.2	58.1
Hospital transferred, %	5.5	11.6	35.7	21.4
*History of comorbidities*				
Cancer, %	3.5	5.4	3.9	5.3
Chronic lung disease, %	13.9	20.5	20.0	18.8
Ischemic heart disease, %	4.4	6.9	7.2	10.0
Diabetes, %	22.4	49.7	55.1	53.5
Cerebral vascular disease, %	2.2	3.8	4.3	4.0
Chronic heart failure, %	6.8	17.7	18.1	17.9
Arrhythmia, %	3.8	6.8	8.3	6.5
Hypertension, %	24.1	36.8	40.8	44.0
Chronic kidney disease, %	2.9	6.1	8.1	5.6
Dementia, %	4.2	4.9	2.2	2.6
Dyslipidaemia, %	14.1	19.8	19.6	20.7
Iron deficiency anaemia, %	6.9	8.6	9.1	8.1
Peripheral vascular disease, %	0.4	0.8	0.5	0.7
Liver cirrhosis, %	0.4	0.6	0.6	0.2

*COVID-19* coronavirus disease 2019, *iMV* invasive mechanical ventilation

^a^The median (interquartile range) prescribed dose of pulse methylprednisolone therapy was 1000 (500–1000) mg per day

Table 2 shows the prevalence of treatment modalities and medications among COVID-19 patients throughout hospital admission. Patients were stratified according to their iMV and steroid pulse therapy status. Patients who did not receive iMV and pulse therapy were less likely to receive other clinical interventions during their admission period. Patients requiring iMV showed a higher prevalence of other clinical interventions such as ICU admission. Among the iMV-receiving patients, pulse therapy recipients were more likely to receive other clinical interventions such as other anti-inflammatory medications, non-ventilation oxygen therapy, antibiotics, and intermediate/higher doses of methylprednisolone.

**Table 2:** Prevalence of other clinical interventions among acute COVID-19 patients with or without invasive mechanical ventilation (iMV) throughout the hospital admission period

Pulse methylprednisolone therapy (≥ 500 mg per day)	iMV-free patients		iMV-receiving patients	
	Not prescribed (*n* = 63,149)	Prescribed (*n* = 1814)	Not prescribed (*n* = 1955)	Prescribed (*n* = 430)
ICU admission, %	3.1	15.3	64.1	54.0
RRT initiation, %	1.0	3.6	11.0	9.3
ECMO initiation, %	0.0	0.8	6.4	6.5
Vasopressin, %	0.4	7.5	53.8	55.6
Blood transfusion, %	0.5	5.1	17.3	18.1
Dexamethasone, %	21.8	47.1	32.6	36.0
Tocilizumab, %	1.5	13.0	12.7	24.0
Ivermectin, %	0.2	0.1	0.0	0.7
Hydroxychloroquine, %	0.2	0.3	0.8	0.5
Remdesivir, %	0.1	0.3	0.1	0.0
Baricitinib, %	3.7	24.6	9.9	31.2
Macrolides, %	3.6	13.9	12.6	23.7
Carbapenems, %	1.0	11.0	25.9	34.4
Required oxygen, %	31.8	85.8	N/A	N/A
NPPV, %	0.0	0.4	1.0	1.2
Intermediate/higher dose of methylprednisolone I.V.^a^, %	2.5	38.8	18.8	52.6
Anti-fungal interventions, %	0.1	2.7	3.5	6.5

*COVID-19* coronavirus disease 2019, *ICU* intensive care unit, *RRT* renal replacement therapy, *ECMO* extracorporeal membrane oxygenation, *NPPV* non-invasive positive pressure ventilation, *I.V.* intravenous, *N/A* not available

^a^Intermediate/higher dose of methylprednisolone I.V. was defined as 40–250 mg per day

The prevalence of treatment modalities and medications before and after methylprednisolone pulse therapy is shown in Table 3. ICU admission, renal replacement therapy, extracorporeal membrane oxygenation, vasopressin, blood transfusion, tocilizumab, baricitinib, macrolides, and carbapenems were more frequently introduced before the initiation of steroid pulse therapy than after pulse therapy. These differences in treatment modalities before and after pulse therapy were larger among iMV-receivers than among iMV-free patients. The findings also showed an increase in oxygen therapy after the initiation of steroid pulse therapy among iMV-free patients and increments in anti-fungal interventions among both iMV-free and iMV-receiving patients (Table 3).

**Table 3:** Prevalence of other clinical interventions before or after the prescription of pulse methylprednisolone therapy among invasive mechanical ventilation (iMV)-receiving and iMV-free acute COVID-19 patients

Timing with pulse methylprednisolone therapy (≥ 500 mg per day)	iMV-free patients (*n* = 64,963)		iMV-receiving patients (*n* = 2385)	
	Prior to prescription	Posterior to prescription	Prior to prescription	Posterior to prescribed
ICU admission, %	5.8	9.5	47.0	7.0
RRT initiation, %	1.3	2.4	8.1	1.2
ECMO initiation, %	0.2	0.6	6.5	0.0
Vasopressin, %	2.8	4.7	54.7	0.9
Blood transfusion, %	3.3	1.8	17.9	0.2
Dexamethasone, %	20.6	26.5	14.4	21.6
Tocilizumab, %	9.7	3.3	21.9	2.1
Ivermectin, %	0.0	0.1	0.5	0.2
Hydroxychloroquine, %	0.1	0.3	0.5	0.0
Remdesivir, %	0.1	0.2	0.0	0.0
Baricitinib, %	16.1	8.5	24.2	7.0
Macrolides, %	7.7	6.3	19.1	4.7
Carbapenems, %	6.9	4.0	32.8	1.6
Required oxygen, %	38.1	47.7	N/A	N/A
NPPV, %	0.4	0.1	1.2	0.0
Intermediate/higher dose of methylprednisolone I.V.^a^, %	8.9	29.9	6.6	46.0
Anti-fungal interventions, %	0.5	2.2	0.3	6.2

*COVID-19* coronavirus disease 2019, *ICU* intensive care unit, *RRT* renal replacement therapy, *ECMO* extracorporeal membrane oxygenation, *NPPV* non-invasive positive pressure ventilation, *I.V.* intravenous, *N/A* not available

^a^Intermediate/higher dose of methylprednisolone I.V. was defined as 40–250 mg per day

### Influence of methylprednisolone pulse therapy on the risk of in-hospital mortality

Table 4 summarises the association between the risk of in-hospital mortality and the use of intravenous methylprednisolone pulse and intermediate/higher dose therapy in patients with acute COVID-19. In the model adjusted for age, sex, and various conventional confounders at baseline, intravenous pulse therapy with methylprednisolone increased the risk of in-hospital mortality among both iMV-free (HR 2.86, 95% CI 2.53–3.22), and no association in iMV-receiving patients (HR 1.01, 95% CI 0.88–1.16). Remarkably, when we controlled for immortal time bias and time-dependent confounders with a marginal structure model, a reduction in the risk of in-hospital mortality with the use of intravenous methylprednisolone pulse therapy was observed in the iMV-receiving group (HR 0.59, 95% CI 0.52–0.68). The impact was greater than that of 40–250 mg (HR 0.80, 95% CI 0.71–0.89). However, the benefits of pulse therapy on the risk of in-hospital mortality were not observed in iMV-free patients; the HR (95% CI) was 3.38 (3.02–3.79) for pulse therapy and was 2.38 (2.11–2.70) for the intermediate/higher dose.

**Table 4:** Hazard ratios (HRs) and 95% confidence intervals (95% CIs) for the risk of in-hospital mortality after pulse methylprednisolone therapy and an intermediate/higher dose of methylprednisolone among acute COVID-19 patients with or without invasive mechanical ventilation (iMV)

	iMV-free patients		iMV-receiving patients	
Pulse methylprednisolone therapy (≥ 500 mg per day)^a^	Not prescribed	Prescribed	Not prescribed	Prescribed
No. at risk	63,149	1814	1955	430
No. of deaths	1441	353	483	123
Crude mortality	2.3%	19.5%	24.7%	28.6%
Multivariable-adjusted^b^ HRs (95% CIs)	Reference	2.86 (2.53–3.22)	Reference	1.01 (0.88–1.16)
Marginal structural model^c^ HRs (95% CIs)	Reference	3.38 (3.02–3.79)	Reference	0.59 (0.52–0.68)

Intermediate/high dose of methylprednisolone (40–250 mg per day)	Not prescribed	Prescribed	Not prescribed	Prescribed
No. at risk	64,500	2253	1790	595
No. of deaths	2012	238	456	150
Crude in-hospital mortality	3.1%	10.6%	26.0%	25.2%
Multivariable-adjusted^b^ HRs (95% CIs)	Reference	1.76 (1.53–2.02)	Reference	0.85 (0.71–1.03)
Marginal structural model^c^ HRs (95% CIs)	Reference	2.38 (2.11–2.70)	Reference	0.80 (0.71–0.89)

*COVID-19* coronavirus disease 2019

^a^The median (interquartile range) prescribed pulse methylprednisolone therapy was 1000 (500–1000) mg per day; the median (interquartile range) intermediate/higher dose of intravenous methylprednisolone was 80 (40–250) mg per day

^b^Multivariable-adjusted models were adjusted to demographic information (age, sex, smoking status, and body mass index), status at hospital admission (coma, shock, cardio-respiratory failure, and transferred hospital), and history of comorbidities (cancer, chronic lung disease, ischemic heart disease, diabetes, cerebral vascular disease, chronic heart failure, arrhythmia, hypertension, chronic kidney disease, dementia, dyslipidaemia, iron deficiency anaemia, peripheral vascular disease, and liver cirrhosis)

^c^Marginal structural model controlled further for the time to other clinical implications (time implicated to intensive care unit administration, renal replacement therapy administration, extracorporeal membrane oxygenation, vasopressin, blood transfusion, dexamethasone, tocilizumab, ivermectin, hydroxychloroquine, remdesivir, baricitinib, macrolides, carbapenems, required oxygen, non-invasive positive pressure ventilation, intermediate/higher doses or pulse methylprednisolone therapy, and anti-fungal interventions)

Table S2 in Additional file 1 shows the comparison of HRs of in-hospital mortality for patients prescribed only intermediate/higher doses of steroids and for patients prescribed only methylprednisolone pulse therapy. The largest reduction in the risk of in-hospital mortality with the use of pulse therapy was observed in the iMV-receiving group (HR 0.49; 95% CI 0.41–0.60) in the marginal structural model compared to that of the patients with intermediate/higher dose of steroids (HR 0.80, 95% CI 0.71–0.93). We confirmed the dose–response association between a higher dose (for 40, 80, 125–250, 500, and ≥ 1000 mg) of methylprednisolone and lower risk of in-hospital mortality among iMV-receiving patients, but not for iMV-free patients (Additional file 1: Fig. S2).

**Fig. 3:**
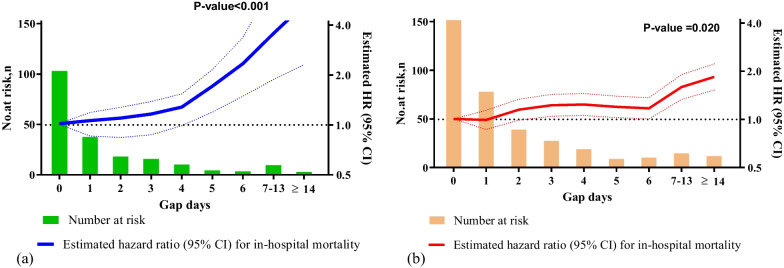
The relative risk of in-hospital mortality according to gap days between the time of intubation of mechanical ventilation and **a** pulse methylprednisolone use (≥ 500 mg per day) or **b** intermediate/higher dose of steroid (40–250 mg per day) among COVID-19 patients, using the restricted subset dataset. No-gap day (day 0) was set as reference to compare risk of in-hospital mortality among gap days both in **a** and **b**. We analysed 66,418 out of 67,348 patients, since 930 patients received both an intermediate/higher dose of intravenous methylprednisolone and pulse methylprednisolone therapy during the same admission period. The median (interquartile range) pulse methylprednisolone therapy was 1000 (500–1000) mg per day. The median (interquartile range) intermediate/higher dose of intravenous methylprednisolone was 80 (40–250) mg per day

Furthermore, a smaller gap (less than five days) between intubation and the initiation of steroid pulse therapy was associated with a lower risk of in-hospital mortality (Fig. 3a); however, this association was weaker with intermediate/higher doses of methylprednisolone (Fig. 3b).

In sensitivity analyses, the results from the time-dependent propensity score sequential matching were not considerably different from the results of primary analyses (Additional file 1: Table S3). In addition, *E*-values, the minimum HRs required to shift the primary association to not significant by unmeasured confounders, were generally higher than primary associations as follows: 6.62 for iMV-free patients treated with steroid pulse therapy, 2.78 for iMV-receiving patients treated with steroid pulse therapy, 4.19 for iMV-free patients treated with the intermediate/higher intravenous steroid therapy, and 1.10 for iMV-receiving patients treated with intermediate/higher intravenous steroid therapy (Additional file 1: Table S4).

## Discussion

We examined the impact of intravenous methylprednisolone pulse therapy on the risk of in-hospital mortality in a nationwide in-hospital clinical cohort. To provide novel evidence for acute COVID-19 patients, our results, which were obtained by controlling immortal time bias and time-dependent confounders, indicated that among iMV-receiving patients, intravenous methylprednisolone pulse therapy with doses of 500–1000 mg per day was significantly associated with a lower risk of in-hospital mortality than in those not treated with steroid pulse therapy or intermediate dose of steroids.

Our results extend the findings of previous studies. First, this study adds insights in the evaluation of the appropriate dosage and timing of steroids among acute COVID-19 patients, after the RECOVERY trial demonstrated the effectiveness of low-dose steroids of dexamethasone 6 mg (converted equity as methylprednisolone of 32 mg) in treating COVID-19 patients who require oxygen therapy [1]. Second, the findings showed that steroid pulse doses may improve survival in limited populations of patients with potentially fatal COVID-19, such as iMV-receivers. Indeed, regardless of steroid dose, there was no improvement among iMV-free patients. Similar to our results, prior studies reported that appropriate selection of patients to be treated with high-dose methylprednisolone is necessary to optimise the risk–benefit balance of steroids for acute COVID-19 patients [7, 25–28].

As for iMV-free acute COVID-19 patients, intravenous methylprednisolone pulse therapy was associated with a higher risk of in-hospital mortality than in those not treated with steroids. The results suggest that a limited population of acute COVID-19 patients would benefit from steroid pulse therapy, similar to previous studies [2, 27, 29]. Additionally, intubation status can be a marker of severe lung inflammation requiring a pulse dose of steroids. Indeed, our study showed that the rates of various other clinical interventions significantly decreased after pulse therapy in iMV-receivers, whereas such reductions were not apparent in iMV-free patients. However, even though we employed a marginal structural model, residual confounder bias from iMV-free patients should be carefully taken into account. In comparison with other groups, iMV-free patients in our cohort without intravenous methylprednisolone pulse therapy had obviously better clinical characteristics such as younger age, healthier at admission, and lower prevalence of comorbidities.

Our study results also indicated that smaller gap days between intubation and pulse therapy (e.g. intervals less than five days) were associated with lower in-hospital mortality among iMV-receiving patients. Few studies have examined the appropriate timing for initiating steroid pulse therapy in acute COVID-19 patients [30, 31]. In one case report of seven intubated acute COVID-19 patients who received the steroid pulse therapy within 0–1 day after intubation, all patients recovered and were extubated [31]. However, the optimal timing of steroid initiation has not been sufficiently examined in quantitative datasets [5, 30]. The present study provides clinically applicable information, particularly regarding the dose and timing of steroid use.

Impacts and clinical consequences of intensive dosing of steroid for hospitalised COVID-19 patients remain uncertain. Moreover, regulations on the upper limit of the steroid dose among hospitalised COVID-19 patients have not been described in international guidelines from the National Institution of Health of the United States [32] and the World Health Organization [33]. Additionally, Japanese clinical practice guidelines for drug management for COVID-19 described benefits of steroid pulse therapy for severe acute COVID-19 patients, despite the insufficiency of scientific evidence [34]. Therefore, our findings, which highlight the benefits and dose–response efficiency of steroid pulse therapy, could improve the current situation.

The strengths of our study include the large sample size and various records of other clinical interventions from a nationwide clinical registry among patients with acute COVID-19. The study settings enabled the examination of recent study objectives. To the best of our knowledge, our study is the first to control for immortal time bias to examine the association between the use of steroids and the risk of in-hospital mortality in acute COVID-19 patients. Moreover, this study could provide a concrete setting and timing for appropriate methylprednisolone pulse therapy in a sufficiently large cohort.

The study also had several limitations. First, this was an observational study influenced by unknown confounders. For example, we could not obtain detailed clinical information such as positive end-expiratory pressure, respiratory rate, PaO_2_/FiO_2_ ratio, use of prone position, and doses of vasopressor agents. Such factors might have confounding effects on the association between the use of steroid pulse therapy and in-hospital mortality. The calculated *E*-value, which indicates the impact of unmeasured confounders and their potential to invalidate the results of our primary target association, was universally high, at 1.81–6.62. Therefore, the unmeasured confounders are not likely to shift our conclusions. Second, because our study was conducted only at acute care hospitals in Japan, the results should be carefully applied for other races, demographics, and countries. For example, (1) the intubation rates of COVID-19 patients (3.5%) are considered to be lower than those in the studies from other countries (4–12%) [35], and (2) 46.4% of iMV-receiving patients were treated in non-ICU settings in Japan [36]. This is primarily because of the unique health policy and special units in addition to ICU in hospitals of Japan [36, 37]. Third, although our data included a large number of acute COVID-19 patients from a quarter of all acute care hospitals in Japan, the results should be carefully interpreted considering that our target population receiving iMV or steroid pulse therapy represents only a small subgroup of all acute COVID-19 patients. Fourth, we could not specifically evaluate the adverse effects of pulse therapy with methylprednisolone, including nosocomial infectious diseases and non-infectious disorders. Fifth, we did not follow up on the impacts following discharge among the survivors. Some in-hospital survivors might have died within a relatively short period after discharge or experienced long-term complications following the use of steroid pulse therapy. Evaluation of longitudinal follow-up data should be performed in future studies to examine the impact of pulse dosing of steroids during the acute phase of COVID-19 infections on post-discharge outcomes.

## Conclusion

Our results obtained by controlling for immortal time bias and time-dependent confounders suggest the dose–response efficiency of methylprednisolone therapy, including steroid pulse therapy, in lowering the risk of in-hospital mortality, particularly among iMV-receiving patients, when they received the pulse soon after (such as < 5 days) iMV initiation. However, the pulse therapy did not reduce the risk of in-hospital mortality among iMV-free patients. These findings could be a major milestone in improving in-hospital mortality among patients with acute and critical COVID-19.

## Supplementary Information

**Additional file 1: **Supplementary Tables and Figures.

**Additional file 2: **Supplementary Text.

## Data Availability

Data have not been shared prior to being thoroughly analysed. All DPC datasets are directed at the Ministry Health and Welfare, Japan.

## Abbreviations

CI: Confidence interval
COVID-19: Coronavirus disease 2019
DPC: Diagnosis and procedure combination
HR: Hazard ratio
ICD-10: International Classification of Diseases and Related Health Problems, 10th Revision
ICU: Intensive care unit
iMV: Invasive mechanical ventilation
MDV: Medical Data Vision
