# Supplementary material for: Intravenous methylprednisolone pulse therapy and the risk of in-hospital mortality among acute COVID-19 patients: Nationwide clinical cohort study
Source: Crit Care. 2023 Feb 8;27:53. doi: 10.1186/s13054-023-04337-5 (PMC9906603; doi:10.1186/s13054-023-04337-5)
Supplement: Supplementary file 1 — Additional file 1: Supplementary Tables and Figures. [file 13054_2023_4337_MOESM1_ESM.docx]

**Additional File 1**

**Figure S1.** Prevalence for each dose of prescribed intravenous methylprednisolone.

**Figure S2.** Forest plot of hazard ratios (HRs) and 95% confidence intervals (95% CIs) for the risk of in-hospital mortality after doses of 40 mg, 80 mg, and 125&250 mg of intravenous methylprednisolone among acute COVID-19 patients with or without invasive mechanical ventilation (iMV). HRs were calculated with marginal structural model analysis and adjusted for demographic information (age, sex, smoking status, and body mass index), status at hospital admission (coma, shock, cardio-respiratory failure, and transferred hospital), history of comorbidities (cancer, chronic lung disease, ischemic heart disease, diabetes, cerebral vascular disease, chronic heart failure, arrhythmia, hypertension, chronic kidney disease, dementia, dyslipidaemia, iron deficiency anaemia, peripheral vascular disease, and liver cirrhosis), and time to other clinical implications (time to intensive care unit admission, renal replacement therapy administration, extracorporeal membrane oxygenation, vasopressin, blood transfer, dexamethasone, tocilizumab, ivermectin, hydroxychloroquine, remdesivir, baricitinib, macrolides, carbapenems, oxygen, non-invasive positive pressure ventilation, intermediate/higher doses or pulse methylprednisolone therapy, and anti-fungal interventions).

**Table S1.** Monthly variation in dose and proportion of prescribed intravenous methylprednisolone in all COVID-19 patients during the study period.

| Prescribed intravenous methylprednisolone | | | |
| --- | --- | --- | --- |
| Date | | Median dose (interquartile range), mg | ％ of prescribed intravenous methylprednisolone |
| 2020 | January | 500 (125–1000) | 7.3% |
|  | February | 750 (500–1000) | 7.0% |
|  | March | 1000 (80–1000) | 6.5% |
|  | April | 125 (80–500) | 5.8% |
|  | May | 1000 (500–1000) | 3.5% |
|  | June | 250 (90–750) | 1.7% |
|  | July | 500 (125–1000) | 1.6% |
|  | August | 125 (80–250) | 3.1% |
|  | September | 500 (80–1000) | 2.5% |
|  | October | 125 (80–1000) | 4.0% |
|  | November | 250 (80–1000) | 3.6% |
|  | December | 500 (120–1000) | 5.5% |
| 2021 | January | 500 (125–1000) | 4.0% |
|  | February | 1000 (125–1000) | 6.5% |
|  | March | 500 (80–1000) | 5.7% |
|  | April | 500 (125–1000) | 4.9% |
|  | May | 500 (125–1000) | 7.1% |
|  | June | 125 (80–1000) | 9.8% |
|  | July | 125 (80–500) | 8.0% |
|  | August | 500 (125–1000) | 4.8% |
|  | September | 250 (120–1000) | 7.4% |
|  | October | 125 (125–500) | 5.9% |
|  | November | 250 (80–500) | 3.0% |
|  | December | N/A | N/A |

COVID-19: Coronavirus disease 2019; N/A: not assessed.

**Table S2.** Comparison of sole use of intravenous methylprednisolone of intermediate/higher dose or pulse therapy and risk of in-hospital mortality among COVID-19 patients with and without a mechanical ventilator.^a^

|  | iMV-free patients | | | iMV-receiving patients | | |
| --- | --- | --- | --- | --- | --- | --- |
| Dose of intravenous methylprednisolone^b^ | Not conducted | Intermediate/higher^c^ (40–250 mg per day) | Pulse therapy  (≥ 500 mg per day) | Not Conducted | Intermediate/higher^c^ (40–250 mg per day) | Pulse therapy  (≥ 500 mg per day) |
| No. at risk | 61,600 | 1,549 | 1,110 | 1,586 | 369 | 204 |
| No. of deaths | 1,345 | 69 | 211 | 401 | 82 | 55 |
| Crude in-hospital mortality | 2.2% | 4.5% | 19.5% | 25.3% | 22.2% | 27.0% |
| Multivariable adjusted^d^ HRs (95% CIs) | Reference | 1.50 (1.22–1.85) | 3.24 (2.79–3.78) | Reference | 0.81 (0.64–1.03) | 1.20 (0.89–1.59) |
| Marginal structural^e^ model HRs (95% CIs) | Reference | 2.96 (2.49–3.52) | 4.86 (4.23–5.58) | Reference | 0.80 (0.71–0.93) | 0.49 (0.41–0.60) |

HR: hazard ratio; 95% CI: 95% confidence interval; COVID-19: coronavirus disease 2019.

^a^ Excluded 930 individuals from original dataset due to conducted both pulse and intermediate/higher dose of intravenous methylprednisolone during hospital admission.

^b^ The median (interquartile range) prescribed pulse methylprednisolone therapy dose was 1000 (500–1000) mg per day.

^c^ The median (interquartile range) intermediate/higher dose of intravenous methylprednisolone was 80 (40–250) mg per day.

^d^ Multivariable adjusted models were adjusted to demographic information (age, sex, smoking status, and body mass index), status at hospital admission (coma, shock, cardio-respiratory

failure, and transferred hospital), and history of comorbidities (cancer, chronic lung disease, ischemic heart disease, diabetes, cerebral vascular disease, chronic heart failure, arrhythmia, hypertension, chronic kidney disease, dementia, dyslipidaemia, iron deficiency anaemia, peripheral vascular disease, and liver cirrhosis).

^e^ Marginal structural model controlled further for time to other clinical implications (time to intensive care unit administration, renal replacement therapy administration, extracorporeal membrane oxygenation, vasopressin, blood transfer, dexamethasone, tocilizumab, ivermectin, hydroxychloroquine, remdesivir, baricitinib, macrolides, carbapenems, oxygen, non-invasive positive pressure ventilation, and anti-fungal interventions).

**Table S3.** Hazard ratios (HRs) and 95% confidence intervals (95% CIs) for the risk of in-hospital mortality after pulse methylprednisolone therapy and intermediate/higher dose of methylprednisolone with time-dependent propensity score matching among acute COVID-19 patients with or without mechanical ventilation (iMV).^a^

|  | iMV-receiving patients | |
| --- | --- | --- |
|  | Intermediate/higher dose of methylprednisolone  (40-250mg per day; n = 1,814 vs. 1,814) | Pulse methylprednisolone therapy  (≥ 500 mg per day; n = 420 vs. 420) |
| Not prescribed (Control), HR (95% CI) | Reference | Reference |
| Prescribed (Case), HR (95% CI) | 0.64 (0.52–0.80) | 0.37 (0.24–0.57) |
|  | iMV-free patients | |
|  | Intermediate/higher dose of methylprednisolone  (40-250mg per day; n = 2,253 vs. 2,253) | Pulse methylprednisolone therapy  (≥ 500 mg per day; n = 583 vs. 583) |
| Not prescribed (Control), HR (95% CI) | Reference | Reference |
| Prescribed (Case), HR (95% CI) | 2.87 (1.97–4.17) | 3.96 (3.04–5.15) |

^a^ Time-dependent propensity score matching was examined with demographic information (age, sex, smoking status, and body mass index), status at hospital admission (coma, shock, cardio-respiratory failure, and transferred hospital), history of comorbidities (cancer, chronic lung disease, ischemic heart disease, diabetes, cerebral vascular disease, chronic heart failure, arrhythmia, hypertension, chronic kidney disease, dementia, dyslipidaemia, iron deficiency anaemia, peripheral vascular disease, and liver cirrhosis), and time to other clinical implications (time to intensive care unit administration, renal replacement therapy administration, extracorporeal membrane oxygenation, vasopressin, blood transfer, dexamethasone, tocilizumab, ivermectin, hydroxychloroquine, remdesivir, baricitinib, macrolides, carbapenems, oxygen, non-invasive positive pressure ventilation, and anti-fungal interventions)..

^b^ The median (interquartile range) prescribed pulse methylprednisolone therapy dose was 1000 (500–1000) mg per day; the median (interquartile range) intermediate/higher dose of intravenous methylprednisolone was 80 (40–250) mg per day.

**Table S4.** The minimum required *E*-value in primary analysis

|  | Pulse methylprednisolone therapy (≥500 mg per day) | | Intermediate/high dose of methylprednisolone (40–250 mg per day) | |
| --- | --- | --- | --- | --- |
| Subjects | iMV-free | iMV-receiving | iMV-free | iMV-receiving |
| MSM-HR | 3.38 | 0.59 | 2.38 | 0.80 |
| *E*-value^a^, HR | 6.62 | 2.78 | 4.19 | 1.10 |

^a^ The *E*-value is the minimum required HR that unmeasured confounders need to move the MSM-HRs for the risk of in-hospital mortality in Table 4 to null.

iMV: invasive mechanical ventilation; MSM: marginal structural model; HR: hazard ratio
