# Supplementary material for: Intravenous methylprednisolone pulse therapy and the risk of in-hospital mortality among acute COVID-19 patients: Nationwide clinical cohort study
Source: Crit Care. 2023 Feb 8;27:53. doi: 10.1186/s13054-023-04337-5 (PMC9906603; doi:10.1186/s13054-023-04337-5)
Supplement: Supplementary file 2 — Additional file 2: Supplementary Text. [file 13054_2023_4337_MOESM2_ESM.docx]

**Additional File 2**

**Supplementary Text.** Construction of the marginal structure model analysis

We used cloning, censoring, and weighting to construct a weighted marginal structural model, which enables elimination of immortal time bias in the estimation and comparison of relative risk^1–3^.

In step one, we created a clone of each original patient (original patient:clone = 1:1) and assigned the clone to the opposite treatment arm from that of the original patient. For example, if the original patient received pulse therapy, the clone was assigned to not receive pulse therapy, and vice versa. In step two, we censored follow-up of original patients and their according to the date of discharge, death, or final follow-up at 31 December 2021. Additionally, only clones were censored at the date when the corresponding original patients received pulse therapy. Although such censoring is likely to be informative, it would still lead to selection bias, including immortal time bias, lead time bias, and survivor bias. Therefore, in step three, we (1) calculated the individual probability weights of not being censored for each day based on patient demographics, history of comorbidities, hospital admission status, and other time-varying clinical interventions; and (2) examined hazard ratios using weighted marginal structural models.

To estimate the probability of each patient not being censored until day *X*, the estimated probabilities of not being censored on day *X – 1* was multiplied by that at *X*. We used the stabilised inversed probability weight **(Figures 1–2)**. Stabilised inversed probability weighting enables producing narrower confidence intervals along with better coverage rates than non-stabilised weights^4^. Stabilised inversed probability weights were derived using the probability for time to not being censored and time to pulse methylprednisolone therapy. The probability of pulse methylprednisolone therapy was examined using the same approach described above. Further, we truncated two side outliers of probability weights close to mean probability weight of 1.00. ^5^ The probability weights higher than the 99th percentile value were fixed at the value of the 99th percentile, whereas weights lower than the 1st percentile value were fixed at the value of the 1st percentile. Finally, we included the stabilised inversed probability weight to the Cox proportional regression model based on the assessment for pulse methylprednisolone therapy and risk of in-hospital mortality.

References:

1 Hernán MA. How to estimate the effect of treatment duration on survival outcomes using observational data. *BMJ* 2018; **360**: k182.

2 Hernán MA, Robins JM. Using Big Data to Emulate a Target Trial When a Randomized Trial Is Not Available. *Am J Epidemiol* 2016; **183**: 758–64.

3 Cotton CA, Heagerty PJ. *https://doi.org/101214/14-AOAS774* 2014; **8**: 2356–77.

4 Robins JM, Hernán MÁ, Brumback B. Marginal structural models and causal inference in epidemiology. *Epidemiology* 2000; **11**: 550–60.

5 Cole SR, Hernán MA. Constructing Inverse Probability Weights for Marginal Structural Models. *Am J Epidemiol* 2008; **168**: 656–64.
